# Development and Psychometric Properties of the *Questionnaire for Assessing Educational Podcasts* (QAEP)

**DOI:** 10.3389/fpsyg.2020.579454

**Published:** 2020-11-23

**Authors:** Rafael Alarcón, María J. Blanca

**Affiliations:** Department of Psychobiology and Behavioral Sciences Methodology, University of Málaga, Málaga, Spain

**Keywords:** podcasts, cross-validation, reliability analysis, validity evidence, feedback

## Abstract

The aim of this research was to develop and validate the *Questionnaire for Assessing Educational Podcasts* (QAEP), an instrument designed to gather students’ views about four dimensions of educational podcasts: access and use, design and structure, content adequacy, and value as an aid to learning. In study 1 we gathered validity evidence based on test content by asking a panel of experts to rate the clarity and relevance of items. Study 2 examined the psychometric properties of the QAEP, including confirmatory factor analysis with cross-validation to test the factor structure of the questionnaire, as well as item and reliability analysis. The results from study 1 showed that the experts considered the items to be clearly worded and relevant in terms of their content. The results from study 2 showed a factor structure consistent with the underlying dimensions, as well as configural and metric invariance across groups. The item analysis and internal consistency for scores on each factor and for total scores were also satisfactory. The scores obtained on the QAEP provide teachers with direct student feedback and highlight those aspects that need to be enhanced in order to improve the teaching/learning process.

## Introduction

The incorporation of information and communication technologies (ICT) into the educational field over the last decade has produced important changes in the teaching/learning process. Among these technologies, podcasts have become increasingly popular. Podcasts are digital media files comprising audio and/or video which can be automatically downloaded from the web to devices such as smartphones, PCs or MP3/4 players (O’Bannon et al., 2011; Alarcón et al., 2017; O’Connor and Andrews, 2018). Their ease of use without restrictions of time and place, coupled with rapid and free availability for most portable devices, makes them a useful tool for promoting cooperative and self-directed learning (Association of College and Research Libraries, 2000; Evans, 2008; Heilesen, 2010; Hill and Nelson, 2011; Reychav and Wu, 2015; Hargett, 2018; O’Connor et al., 2020a, b), especially in higher education (Vajoczki et al., 2010; Alarcón et al., 2017).

Research shows that podcasts have been used in a wide variety of ways in higher education (McGarr, 2009; Chester et al., 2011; Van Zanten et al., 2012; Popova et al., 2014). These include: (a) recording of face-to-face lectures that can then be used as substitutes for traditional classes or as a supplementary form of content review (Gosper et al., 2007; Lightbody et al., 2007; William and Fardon, 2007; McGarr, 2009; McKenzie, 2008; Van Zanten, 2008; Han and Klein, 2019); (b) creative or student-generated podcasts, which have attracted particular attention among researchers as they give students an active role in the learning process, helping to develop competences such as critical thinking (Frydenberg, 2008), collaborative knowledge (Lee et al., 2008), and teamwork and technological skills (Cane and Cashmore, 2008); (c) tutorials (Tynan and Colbran, 2006), showing the steps involved in a specific activity, and glossaries of key terms (Lightbody et al., 2007), which introduce key concepts of a subject; and (d) short 3–5 min podcasts, which are becoming very popular as a way of summarizing a lecture or presenting basic concepts (Lee and Chan, 2007; Abdous et al., 2012; Van Zanten et al., 2012), and which are usually used as complementary material (Bell et al., 2007; Alarcón et al., 2017; Han and Klein, 2019; Matulewicz et al., 2020).

The empirical evidence about the effectiveness and benefits of podcasting in the educational field is wide ranging. Some researchers have focused on the impact that the use of this technology has on cognitive and affective variables related to intellectual and emotional experiences, such as learning, academic performance, comprehension and anxiety, among others (Khechine et al., 2013). The results indicate that students report an improvement in their learning, better academic performance and comprehension of content, less anxiety, and greater commitment to study (Lee and Chan, 2007; Morris, 2010; Hill et al., 2012; Kennedy and Thomas, 2012; Popova et al., 2014; Rockhill et al., 2019).

Student satisfaction with podcasts is another indicator used to assess the effectiveness of this tool (Vajoczki et al., 2010). Student satisfaction refers to the “favourability of a student’s subjective evaluation of the various outcomes and experiences associated with education” (Elliott and Shin, 2002: 198). In order to evaluate this variable, Alarcón et al. (2017) designed the *Student Satisfaction with Educational Podcasts Questionnaire* (SSEPQ). This questionnaire consists of 10 Likert-type items, each with four response options, which assess satisfaction with regard to perceived content adequacy, ease of use, usefulness and benefits to learning from the podcast. The construct validity analysis indicated a single factor, such that the total score on the questionnaire provides a general index of students’ satisfaction with podcasts. As part of the validation study the authors applied the questionnaire to psychology undergraduates and found that they were highly satisfied with the use of short 3–5 min podcasts in a Methods and Statistics course (Alarcón et al., 2017).

The main advantage of the SSEPQ is its brevity and its ability to provide teachers with direct and general feedback from students. However, it does not consider specific aspects related to technical features, content adequacy or the design and structure of podcasts. Some authors have emphasized the importance of evaluating the information available through media, but have also pointed out the lack of agreement regarding quality indicators for assessing podcasts (Paterson et al., 2015; Singh et al., 2016; Kaahwa et al., 2019). Although studies have identified a variety of indicators, related mainly to credibility, content or design (Lin et al., 2015; Paterson et al., 2015; Thoma et al., 2015), these indicators were designed for external peer or expert assessment, rather than for evaluation by podcast users, such as students. Consequently, there is need for a questionnaire that assesses the educational podcasts used by students, and which could provide teachers with useful information for improving their learning tools. The purpose of this study was to develop and to examine the psychometric properties of the *Questionnaire for Assessing Educational Podcasts* (QAEP), an instrument that considers four dimensions of these podcasts: access and use, design and structure, content adequacy, and value as an aid to learning. The research involved two studies. In study 1 we gathered validity evidence based on test content (content validity) by asking a panel of experts to rate the clarity and relevance of items. Study 2 involved a comprehensive psychometric analysis of the QAEP, including confirmatory factor analysis (CFA) with cross-validation to examine the factor structure of the questionnaire, as well as calculation of corrected item-factor correlations and corrected item-total correlations and reliability testing. Finally, we analyzed and interpreted the scores obtained when administering the questionnaire to a sample of psychology undergraduates.

## Study 1: Design of the Questionnaire and Validity Evidence Based on Test Content

### Design of the Questionnaire

We began by conducting focus groups with students in order to determine the aspects of educational podcasts that should be assessed. This process identified four main dimensions to assess: access and use, design and structure, content adequacy, and value as an aid to learning. Next, we drew up a battery of items as indicators of each dimension, taking into account the rules for writing items (Muñiz and Fonseca-Pedrero, 2019) such as representativeness, relevance, specificity, clarity, brevity, simplicity, and comprehensibility. These items were then submitted to further focus group discussion, this time with a panel of experts who were involved in teaching innovation projects and who had experience of teaching or supporting teaching on measurement and research methods courses for undergraduates.

The initial questionnaire consisted of 24 items with a 4-point Likert-type response format (1: strongly disagree, 4: strongly agree), covering the above mentioned dimensions of educational podcasts: (a) access and use (5 items), referring to the ease in locating and accessing podcasts, as well as their use on different devices and in different places (e.g., *It was easy to access the podcasts; I was able to view the podcasts on different devices*); (b) Design and structure (6 items), that is, whether the display format (audio, video, and design), the synchronization between audio and video and the length of the podcasts are adequate (e.g., *The length of the podcasts is appropriate for understanding their content; The audio and video are properly synchronized*); (c) Content adequacy (6 items), which refers to whether the content is presented clearly and whether the information is accurate and adequately represents the topic being studied (e.g., *The content of the podcasts is relevant to the subject; The examples used in the podcasts are appropriate*); and (d) Value as an aid to learning (7 items), that is, whether the podcasts help to improve understanding and reinforce content, increase students’ motivation to study and encourage independent learning (e.g., *The podcasts were a good aid to learning about the subject; The podcasts gave me a better understanding of the subject content*). Higher scores on these factors indicate greater agreement with the dimension being assessed, that is, that the podcasts have been easy to use and access, have an adequate structure and are well designed, have adequate content and are useful for learning, respectively.

### Procedure

The analysis of validity evidence based on test content was focused on the domain relevance, which refers to the extent to which each item on a test is relevant to the targeted domain (Sireci and Faulkner-Bond, 2014). This requires a panel of expert judges, who rate the relevance of the items according to established criteria (Osterlind, 1989). Here we used the blind protocol, in which the judges are given the items and the domains without any indications of which item is meant to be matched with which domain (Hambleton, 1980; Dunn et al., 1999). The judges rated the relevance of each item to each domain on a 5-point scale (1: low degree of relevance; 5: high degree of relevance). The protocol also incorporated a 5-point scale in order to rate the clarity of the item, with higher scores indicating greater clarity.

Six judges (4 men and 2 women) assessed the relevance of the items to the domains. They were aged between 34 and 55 years (*M* = 44, *SD* = 8.25) and had between 11 and 32 years of professional experience. All six were experts in educational innovation and podcasting and had experience of teaching in higher education. Three judges were lecturers in the Faculty of Education, while the other three were from the Faculty of Psychology and were also experts in measurement and research methodology. None of the authors of the present paper participated as a judge and none of the judges was involved in the course in which the podcasts were used.

The protocol was sent via e-mail to the panel of experts. In order to provide a context for their task, the protocol contained a definition of the test domains, as well as instructions for completing the task. The experts were also asked to provide data on age, gender, professional profile, and years of experience.

### Data Analysis

We calculated the means for item clarity, considering as adequate those items with a score of 3 or more (out of 5).

In order to assess the validity evidence based on test content, we calculated the mean scores for item relevance, along with Aiken’s *V* index (Aiken, 1980, 1985) and its 95% confidence interval. The *V* index summarizes the ratings of item content relevance obtained from a panel of experts and ranges from 0 (disagreement) to 1 (perfect agreement). We considered a *V* index associated with the theoretical dimension of 0.70 as satisfactory (Charter, 2003). Penfield and Giacobbi (2004) proposed the calculation of confidence intervals as a means of testing the null hypothesis that *V* is equal to the pre-established cut-off point. Based on this criterion, items were considered to have an adequate degree of relevance if the *V* index was above this cut-off and the 95% confidence interval did not include the value 0.70.

Data analysis was performed with SPSS v24. The program created by Merino and Livia (2009) was used to compute the confidence intervals of the *V* index.

## Results

Means for item clarity were above 3.30 in all cases, indicating that the experts considered them to be clearly worded. However, four items had a *V* index with a 95% confidence interval that included the value 0.70: *It was easy to see the podcasts*, *V* = 0.75 [0.55–0.88]; *The content of the podcasts is presented in a logical order*, *V* = 0.71 [0.51–0.85]; *The podcasts provide clear information about the topic in question*, *V* = 0.79 [0.59–0.91]; and *The podcasts complement the face-to-face lectures*, *V* = 0.75 [0.55–0.88]. These four items were therefore eliminated. The remaining 20 items yielded a *V* index above 0.70 for the theoretical dimension and the confidence interval did not include this value. The mean relevance rating was also above 4 for all these items, indicating that the experts considered them to be relevant to the theoretical dimension. In addition, all these items had a *V* index below 0.40 on the dimensions to which they did not belong. These 20 items formed the final version of the QAEP. The results are shown in Table 1.

**TABLE 1:** Means for clarity and relevance, and values of the V index and its 95% confidence interval.

Items				95% CI	
	Clarity means	Relevance means	*V*	Lower	Upper
Factor 1. Access and use					
1. It was easy to access the podcasts	4.83	4.83	0.95	0.80	0.99
2. I was able to view the podcasts on different devices (smartphone, PC, etc.)	4.83	4.83	0.95	0.80	0.99
3. I was able to view the podcasts in different places	3.33	4.83	0.95	0.80	0.99
4. The podcasts were easy to find online	4.66	4.83	0.95	0.80	0.99
Factor 2. Design and structure					
5. The length of the podcasts is appropriate for understanding their content	4.33	4.50	0.88	0.72	0.96
6. The design of the podcasts (colors, tables, graphics, etc.) is attractive	4.66	4.66	0.92	0.74	0.98
7. The presentation format of the podcasts is good	4.66	4.66	0.92	0.74	0.98
8. The audio of the podcasts is clear	3.51	4.66	0.92	0.74	0.98
9. The audio and video are properly synchronized	4.33	4.66	0.92	0.74	0.98
Factor 3. Content adequacy					
10. The podcasts provide a good summary of the topic	4.33	4.50	0.88	0.72	0.96
11. The terminology used in the podcasts is appropriate	4.50	4.33	0.83	0.71	0.93
12. The examples used in the podcasts are appropriate	4.50	4.66	0.92	0.74	0.98
13. The content of the podcasts is relevant to the subject	4.66	4.83	0.95	0.80	0.99
Factor 4. Value as an aid to learning					
14. The podcasts were a good aid to learning about the subject	4.83	4.83	0.95	0.80	0.99
15. The podcasts reinforced my understanding of the subject	3.66	4.83	0.95	0.80	0.99
16. The podcasts made the subject more enjoyable	4.66	4.66	0.92	0.74	0.98
17. The podcasts were useful for learning about the subject	4.66	4.83	0.95	0.80	0.99
18. I’m satisfied with the podcasts as a learning tool for this subject	4.66	4.83	0.95	0.80	0.99
19. The podcasts encourage independent learning by students	4.66	4.83	0.95	0.80	0.99
20. The podcasts gave me a better understanding of the subject content	4.33	4.83	0.95	0.80	0.99

## Study 2. Psychometric Analysis of the QAEP

We proceeded to obtain validity evidence based on the instrument’s internal structure. First, we tested the proposed factor structure (construct validity) of four first-order factors and one second-order factor. Subsequently, we carried out an analysis of items and of the reliability of the factors and total score. Finally, we performed descriptive analyses of QAEP scores.

## Materials and Methods

### Sample

Participants were 245 students (68 males and 177 females) aged between 18 and 54 years (*M* = 21.22, *SD* = 6.32) who were enrolled in the Research Methods and Statistics course that is offered during the first year of the Degree in Psychology at the University of Malaga (Spain). They all used the podcasts at least once during the academic year. In order to analyze the factor structure the total sample was split into two randomized sub-samples: the first (the calibration sample) consisted of 136 individuals (35 males and 101 females) with an average age of 22.57 years (*SD* = 7.72), while the second (the validation sample) comprised 109 individuals (33 males and 76 females) with an average of 22.06 years (*SD* = 7.17).

### Instrument

The QAEP described in study 1 was administered. We expected the QAEP to show four first-order factors, in accordance with its theoretical dimensions (access and use, design and structure, content adequacy, and value as an aid to learning), and one second-order factor that subsumes these factors and which supports the use of a total score for the assessment of educational podcasts.

For this second study we used 11 educational podcasts related to different topics covered by the aforementioned Research Methods and Statistics course. The podcasts were designed by the authors and created in audio/video format using Microsoft PowerPoint, Audacity^®^ and Camtasia^®^ software. Each podcast presented theoretical and practical content related to the main topics covered by the course syllabus (e.g., introduction to statistical inference, Type I and Type II error, parametric and non-parametric tests, etc.), the purpose being to provide complementary material. The length of the podcasts was 3–5 min, beginning with a short summary of the contents, followed by a step-by-step guide to performing statistical analysis with IBM SPSS and how to interpret the results obtained.

Educational podcasts were uploaded to the course’s virtual campus and were available to students throughout the semester. They were also used in practical classes during the course, thus ensuring that all participants engaged with them at least once during the academic year.

### Procedure

The QAEP was administered to students on the day of the final exam of the Research Methods and Statistics course. Participants were asked to provide basic personal data (code number, age, and gender) and were informed that all data were anonymous and would be used exclusively for research purposes.

### Data Analysis

The internal structure of the QAEP (construct validity) was analyzed by means of CFA. A cross-validation strategy was employed using the two randomized samples described above. In the calibration sample (*n* = 136) we tested a model comprising four first-order factors and one second-order factor. To verify the factor structure underlying the QAEP we then checked the fit of this model in the validation sample (*n* = 109), applying several measures of covariance structure equivalence. Configural invariance was examined to establish whether the number of factors and factor-loading patterns were the same across groups, constraining the factor structure to be equal across the two groups (configural model). We then examined metric invariance in order to test equality with respect to the first-order and second-order factor loadings across groups. This analysis was carried out in two steps: in the first, the model was tested by constraining all first-order factor loadings to be equal, while in the second the model was tested by constraining second-order factor loadings to be equal across groups.

Confirmatory factor analyses were performed via structural equation modeling, using the EQS 6.3 software package (Bentler, 2006) with the maximum likelihood and robust estimation methods and the polychoric correlation matrix of items. The Satorra-Bentler chi-square ($\chi_{S-B}^{2}$) was computed with the following goodness-of-fit indices (Bentler, 2006): the non-normed fit index (NNFI; Bentler and Bonett, 1980), the comparative fit index (CFI; Bentler, 1990), the root mean square error of approximation (RMSEA; Browne and Cudeck, 1993; Steiger, 2000) and the 90% confidence interval for the RMSEA. Values of the NNFI and CFI close to or greater than 0.95 are indicative of a good fit (Hu and Bentler, 1999). Values of the RMSEA less than 0.08 indicate a reasonable fit (Browne and Cudeck, 1993) and those less than 0.06 represent a good fit (Hu and Bentler, 1999). Because the chi-square test to compare the fit of the nested models is sensitive to sample size, the configural and metric invariance was assessed by comparing the CFI values, as recommended by Cheung and Rensvold (2002). It is considered that the constraints are tenable if the decrease in CFI is less than or equal to 0.01 between the most constrained model and the configural model.

Next, we carried out item analyses by computing corrected item-factor correlations and corrected item-total correlations. The score of each respective item was eliminated when computing the corresponding corrected correlation. Values greater than 0.30 are considered satisfactory (De Vaus, 2002). We also obtained evidence of reliability (internal consistency) by calculating Cronbach’s alpha coefficients for scores on each factor and the total score on the QAEP.

Finally, a descriptive analysis was carried out in order to obtain mean scores for each dimension of the QAEP, thus providing a measure of students’ views about the podcasts. The factor score was calculated by summing scores for the items that load on each factor, while the total score was calculated as the sum of all 20 item responses. Item, reliability and descriptive analyses were all computed using IBM SPSS v24.

## Results

The factor structure of the QAEP based on four first-order factors and one second-order factor was tested in the calibration sample and the validation sample. The results showed a good fit in both samples. We then calculated the goodness-of-fit indices related to the test for multigroup configural invariance, all of which indicated a good fit. The goodness-of-fit indices related to the equality of the first-order and second-order factor loadings were also satisfactory. Furthermore, the CFI showed no decrease from the configural model to the model with first-order and second-order factor loadings constrained to be equal across groups. These results indicate that the QAEP has a stable structure across groups. Table 2 shows the fit indices for the cross-validation strategy and Table 3 the factor loadings, all of which are statistically significant.

**TABLE 2:** Fit indices for the second-order factor model of the QAEP for the calibration and validation samples and measurement invariance tests across samples.

Model	$\chi_{S-B}^{2}$	*df*	CFI	NNFI	RMSEA	Δ CFI
Calibration sample	180.08	161	0.997	0.996	0.030 [0.001–0.051]	
Validation sample	170.18	161	0.998	0.998	0.023 [0.001–0.050]	
Configural invariance	358.82	330	0.997	0.997	0.027 [0.001–0.044]	
Equality constraints on first-order factor loadings	366.87	346	0.998	0.998	0.022 [0.001–0.041]	0.001
Equality constraints on first- and second-order factor loadings	312.88	342	0.999	0.999	0.015 [0.001–0.034]	0.001

*Calibration sample, n = 136; validation sample, n = 109; $\chi_{S-B}^{2}$ = Satorra-Bentler chi-square; df = degrees of freedom; CFI = comparative fit index; NNFI = non-normed fit index; RMSEA = root mean square error of approximation with 90% confidence interval; Δ CFI = CFI Configural invariance model – CFI more constrained model.*

**TABLE 3:** Factor loadings, corrected item-factor correlations, item-total correlations and Cronbach’s alpha coefficient for each factor.

QAEP items	Factor loading	Second-order factor loading	Item-factor correlation	Item-total correlation	Cronbach’s alpha
Factor 1. Access and use		0.91			0.75
Item 1. It was easy to access the podcasts	0.92		0.49	0.64	
Item 2. I was able to view the podcasts on different devices (smartphone, PC, etc.)	0.49		0.55	0.36	
Item 3. I was able to view the podcasts in different places	0.57		0.60	0.44	
Item 4. The podcasts were easy to find online	0.88		0.59	0.67	
Factor 2. Design and structure		0.89			0.76
Item 5. The length of the podcasts is appropriate for understanding their content	0.66		0.50	0.54	
Item 6. The design of the podcasts (colors, tables, graphics, etc.) is attractive	0.82		0.61	0.67	
Item 7. The presentation format of the podcasts is good	0.83		0.63	0.57	
Item 8. The audio of the podcasts is clear	0.57		0.45	0.42	
Item 9. The audio and video are properly synchronized	0.66		0.47	0.55	
Factor 3. Content adequacy		0.95			0.80
Item 10. The podcasts provide a good summary of the topic being addressed	0.74		0.60	0.60	
Item 11. The terminology used in the podcasts is appropriate	0.81		0.63	0.65	
Item 12. The examples used in the podcasts are appropriate	0.78		0.61	0.61	
Item 13. The content of the podcasts is relevant to the subject	0.86		0.63	0.66	
Factor 4. Value as an aid to learning		0.83			0.91
Item 14. The podcasts were a good aid to learning about the subject	0.89		0.82	0.75	
Item 15. The podcasts reinforced my understanding of the subject	0.81		0.73	0.67	
Item 16. The podcasts have made the subject more enjoyable	0.63		0.57	0.51	
Item 17. The podcasts were useful for learning about the subject	0.93		0.83	0.77	
Item 18. I’m satisfied with the podcasts as a learning tool for this subject	0.88		0.79	0.76	
Item 19. The podcasts encourage independent learning by students	0.70		0.62	0.56	
Item 20. The podcasts gave me a better understanding of the subject content	0.92		0.82	0.75	

Table 3 also shows the corrected item-factor correlations and corrected item-total correlations, with all values being above 0.30. The Cronbach’s alpha coefficients for scores on each factor were above 0.70, and the value of alpha for the total score was 0.92.

Having verified that the QAEP had adequate psychometric properties, we then performed a descriptive analysis in order to obtain a measure of students’ views about the educational podcasts used in the Research Methods and Statistics course. Table 4 shows the descriptive statistics obtained. Means for all items were above 3 (out of 4).

**TABLE 4:** Mean (M) and standard deviation (SD) for items, factors and total score of the QAEP.

QAEP items	*M*	*SD*
Factor 1. Access and use	14.33	2.01
Item 1. It was easy to access the podcasts	3.84	0.49
Item 2. I was able to view the podcasts on different devices (smartphone, PC, etc.)	3.27	0.83
Item 3. I was able to view the podcasts in different places	3.46	0.72
Item 4. The podcasts were easy to find online	3.76	0.58
Factor 2. Design and structure	17.47	2.30
Item 5. The length of the podcasts is appropriate for understanding their content	3.51	0.65
Item 6. The design of the podcasts (colors, tables, graphics, etc.) is attractive	3.38	0.66
Item 7. The presentation format of the podcasts is good	3.53	0.59
Item 8. The audio of the podcasts is clear	3.41	0.73
Item 9. The audio and video are properly synchronized	3.62	0.58
Factor 3. Content adequacy	14.60	1.82
Item 10. The podcasts provide a good summary of the topic being addressed	3.57	0.61
Item 11. The terminology used in the podcasts is appropriate	3.63	0.60
Item 12. The examples used in the podcasts are appropriate	3.64	0.56
Item 13. The content of the podcasts is relevant to the subject	3.76	0.51
Factor 4. Value as an aid to learning	24.16	3.76
Item 14. The podcasts were a good aid to learning about the subject	3.44	0.65
Item 15. The podcasts reinforced my understanding of the subject	3.46	0.67
Item 16. The podcasts made the subject more enjoyable	3.20	0.77
Item 17. The podcasts were useful for learning about the subject	3.53	0.66
Item 18. I’m satisfied with the podcasts as a learning tool for this subject	3.45	0.64
Item 19. The podcasts encourage independent learning by students	3.54	0.62
Item 20. The podcasts gave me a better understanding of the subject content	3.54	0.62
Total score	70.56	8.16

## Discussion

This paper describes the design and initial evaluation of the *Questionnaire for Assessing Educational Podcasts* (QAEP) (study 1), followed by an exhaustive analysis of its psychometric properties (study 2). The questionnaire comprises 20 items covering four dimensions of educational podcasts: access and use, design and structure, content adequacy, and value as an aid to learning.

Validity evidence based on test content was obtained by asking a panel of experts to assess the clarity and relevance of items. To this end we used the blind protocol, such that the judges did not know which items were matched to which theoretical domain (Hambleton, 1980; Dunn et al., 1999). This approach is considered superior to the non-blind protocol. Based on the results we eliminated four items that did not fulfill the inclusion criterion. The remaining 20 items that formed the QAEP were consistent with the theoretical dimension and also relevant in terms of their content. This indicates that the items of the QAEP adequately represent the dimensions underlying the questionnaire. The experts also considered that these items were clearly worded.

In accordance with the proposed theoretical structure we then used CFA and a cross-validation strategy to test a model based on four first-order factors and one second-order factor. The results showed that the proposed structure fitted the data adequately and that the QAEP had a stable structure across groups, with configural and metric invariance. The second-order factor supports the use of a total score as a measure of students’ views about educational podcasts.

Finally, we conducted a reliability analysis. Cronbach’s alpha coefficients for scores on each factor were above 0.70, and the value of alpha for the total score was 0.92. The corrected item-factor correlations and corrected item-total correlations were also satisfactory.

Having confirmed that the QAEP shows adequate psychometric properties, we then obtained descriptive data for each of its dimensions, administering the questionnaire to a sample of psychology undergraduates. Regarding the dimension *access and use*, the mean score of 14.33 (out of 16) indicates that students considered the podcasts to have been easy to use and access, and that they could use them in different places and on different devices. This reflects a recognized advantage of podcasts as educational tools, namely the possibility of viewing them as often as is wished and on any device (Evans, 2008; Heilesen, 2010; Vajoczki et al., 2010; Hill and Nelson, 2011; Reychav and Wu, 2015; Alarcón et al., 2017). In relation to the dimension *design and structure*, the mean score of 17.47 (out of 20) indicates that students were positive about the length of the podcasts and the display format (audio, video, and design). The highest rating (mean of 14.60 out of 16) was obtained for the *content adequacy* dimension. This indicates that the podcasts were considered to be clear in their presentation and that the information they contained was accurate and provided an adequate summary of the topics addressed. Finally, the mean score of 24.16 (out of 28) on the *value as an aid to learning* dimension suggests that students felt that the podcasts had facilitated their learning of the subject, reinforcing their understanding, increasing their motivation to study and encouraging independent learning. The mean total score of 70.56 (out of 80) likewise shows that the students were very positive about the educational podcasts.

The limitations of this study are that all the participants were psychology undergraduates on a Research Methods and Statistics course and that the evaluation was based on just 11 podcasts used during this course. This may restrict the generalizability of results. Future studies should therefore analyze the applicability of the QAEP to different courses and disciplines (e.g., engineering, medicine, etc.), as well as to different educational levels such as baccalaureate or secondary school.

## Conclusion

The QAEP is a short and easy-to-administer questionnaire for exploring students’ views about educational podcasts, specifically as regards ease of access and use, structure and design, content adequacy, and value as an aid to learning. The scores obtained provide teachers with direct student feedback and highlight those aspects that need to be enhanced in order to improve the teaching/learning process.

## Data Availability Statement

All datasets presented in this study are included in the article/supplementary material.

## Ethics Statement

The studies involving human participants were reviewed and approved by the Experimentation Ethics Committee of University of Malaga. The patients/participants provided their written informed consent to participate in this study.

## Author Contributions

All authors listed have made a substantial, direct and intellectual contribution to the work, and approved it for publication.

## Conflict of Interest

The authors declare that the research was conducted in the absence of any commercial or financial relationships that could be construed as a potential conflict of interest.
